# Powered-gliding/climbing flight performed by bats for saving fuel

**DOI:** 10.1242/bio.061779

**Published:** 2025-09-01

**Authors:** Gottfried Sachs

**Affiliations:** TUM Senior Excellence Faculty, Technical University of Munich, Arcisstraße 21, 80333 München, Germany

**Keywords:** Powered glide, Flight with continual altitude changes, Minimum-fuel flight, Fuel saving

## Abstract

Results of recent research show that bats perform flights with continual altitude changes rather than flying at a constant altitude. However, the current state of knowledge suggests that the reason for these altitude changes is not known, and it is stated in the literature that further study is necessary in order to understand this behaviour. The goal of this paper is to provide an explanation by showing that flights with continual altitude changes constitute a fuel-saving flight mode in bats. The descents in the altitude changes – which were analysed using flight measurement data – show a power support by flapping the wings to yield a powered glide. Accordingly, this flight mode may be termed powered-gliding/climbing flight. Corresponding to the described flight characteristics, powered-gliding/climbing flight can be seen as an extension of flap-gliding flight, which is a flight mode known in the research on animal flight. This paper shows that the powered glide enables a decrease in aerodynamic drag, as well as an explanation of the underlying physical mechanism. I also developed a flight mechanics model of powered/gliding climbing flight in bats. Results based on this model show that fuel consumption can be reduced. Thus, a substantial fuel saving can be achieved when compared with the best flight at constant altitude, which is classically considered as the flight mode requiring the lowest fuel consumption.

## Teaser

**Summary:** A solution is presented to explain why bats perform flights with continual altitude changes. It is shown that this flight mode, termed powered-gliding/climbing flight, enables a fuel saving.

## INTRODUCTION

The energy cost of flight is a basic subject in the flight performance of bats (Norberg, 1990). According to classical theory, flights with minimum fuel consumption are of steady-state nature in the form of a cruise type of flight (Pennycuick, 1975). This type of flight implies that the altitude is constant.

Recent research has revealed that there are flights in bats that show continual altitude changes, comprising of a succession of ascents and descents of varying magnitude (O'Mara et al., 2019a; Roeleke et al., 2018; Cvikel et al., 2015). These findings, which have been made feasible with the availability of appropriate light-weight measurement devices, have led to new insights into the flight behaviour of bats, but have also caused new issues and questions.

These issues and questions have given rise to differing discussions and considerations (Cvikel et al., 2015; O'Mara et al., 2019a,b, 2021; Roeleke et al., 2018; Voigt et al., 2019). In regards to this paper, there are two basic aspects: the reasons why bats carry out altitude changes, and the manner in which the descents of the altitude changes are performed.

Concerning the first aspect, various arguments are discussed and different explanations are presented. One point is that bats made use of mountain slopes to ascend to higher altitudes (Roeleke et al., 2018). Another consideration is related to foraging efforts that are regarded to be greater in flights with large altitude changes (O'Mara et al., 2019a). A further point is that bats use uplift effects associated with the terrain for ascending (O'Mara et al., 2021). The current state of research suggests that there is a lack of knowledge concerning flights with continual altitude changes. That manifests most notably in statements in related papers that future research on this issue is necessary. Cvikel et al. (2015), noted that further study is required to understand this behaviour. Another paper (O'Mara et al., 2019b) addresses this gap in knowledge with the statement that unexpected altitudinal changes were most interesting, and resembled those found in migrating thrushes. Furthermore, it is stated in Voigt et al. (2019) that short altitudinal flights with fast ascents and descents may present a so far unrecognized behaviour in bats, which may be more widespread among other aerial insectivores.

The second aspect is central to the problem under consideration. This is because bats show descents where they flap their wings rather than glide with a fixed wing posture. There are indications that bats perform powered flight in descents and not gliding (Roeleke et al., 2018). Furthermore, it has been found that wingbeat frequency was independent from climbing or descending flight (O'Mara et al., 2019a), implying that these descents showed wing flapping. Another point is that there were unexpected altitudinal changes similar to those of migrating thrushes (O'Mara et al., 2019b; Bowlin et al., 2015). Thus, it can be concluded that there is a spectrum of descents in bats that show flapping of the wings.

The central importance of descents with flapping of the wings concerns the effect that flapping has on drag; flapping causes additional drag (Klein Heerenbrink et al., 2015; Klein Heerenbrink and Hedenström, 2023; Sachs, 2015a; Currie et al., 2023), with a resulting fuel cost. By contrast, a gliding descent with a fixed wing posture, does not incur a fuel cost. Comparing both kinds of descents, it would seem that there is a cost disadvantage to the descents with flapping. This begs the question of why bats nonetheless perform descents with flapping and seem to accept the associated fuel cost.

The goal of this paper is to show that powered-gliding/climbing flight is a flight mode performed by bats. Evidence for the existence of powered glides that are an essential element of this flight mode is provided by analysing flight data. A flight mechanics model has been developed for powered-gliding/climbing flight so that the fuel consumption can be determined. The results achieved with this model show that powered-gliding/climbing flight yields a significant fuel saving when compared with the best flight at constant altitude, which is classically considered to be the flight mode requiring the lowest fuel consumption.

## RESULTS

### Biological aspects of bat flight

Biological aspects of bat flight that are related to this paper are described. This concerns the unique biological abilities of bat wings for generating aerodynamic forces. Bats show unique biological features regarding aerodynamics as they have compliant wings, including specialized muscles (Hedenström and Johansson, 2015a). The wings are formed by a skin membrane that is a highly elastic structure (Norberg, 1976). This can be controlled to change the aerodynamic characteristics of the wing by adjusting the camber of the wing profile. Furthermore, it gives bats the ability to change the shape of their wings and provides a morphing ability (Hedenström and Johansson, 2015a). Compared with birds, bat wings are compliant and have wider range of possible morphological adjustments, implying an ability to control wing morphology according to the aerodynamic demands (Hedenström and Johansson, 2015b).

The flapping motion of the wings and the airspeed yield an effective speed of the airflow over the wing at a given angle of attack in the flight of bats. These properties, together with the morphology, e.g. size and cross-sectional shape (thickness, camber) of the wing, determine the overall aerodynamic force acting on the bat (Hedenström and Johansson, 2015b; Lindhe Norberg et al., 2000). The overall aerodynamic force includes the aerodynamic lift, *L*, the aerodynamic drag, *D*, and the thrust, *T*.

The lift can be expressed as (Norberg, 1990; Rayner, 2001):
(1)

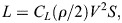
where *C*_*L*_ is the non-dimensional lift coefficient, *ρ* is the air density, *S* is the wing reference area (wing surface) and *V* is the airspeed. Characteristics of the wing, such as thickness, camber and surface texture are included in the lift coefficient, which is a measure of the capacity of the wing to generate lift (Hedenström and Johansson, 2015b).

The total aerodynamic drag *D* experienced by the bat is made up of three components, the induced drag *D*_*i*_, the profile drag *D*_*pro*_ and the parasite drag *D*_*par*_ (Lindhe Norberg et al., 2000; von Busse et al., 2014), yielding:
(2rma)

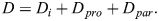


The induced drag *D*_*i*_ is associated with lift generation. The profile drag *D*_*pro*_ is the friction drag of the wings, and the parasite drag *D*_*par*_ is the drag of the body, not including the wings (Lindhe Norberg et al., 2000; Pennycuick, 2008).

The induced drag is given by:
(2rmb)

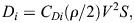
where *C*_*Di*_ is the non-dimensional induced drag coefficient (Norberg, 1990). Accordingly for the profile drag and the parasite drag:
(2rmc)

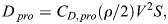

(2rmd)

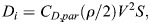
where *C*_*D*,*pro*_ is the profile drag coefficient and *C*_*D*,*par*_ is the parasite drag coefficient (Lindhe Norberg et al., 2000).

The thrust, *T*, is generated by inclining the lift vector forward in the downstroke (Norberg, 1990). The upstroke, which is not considered in this basic treatment, shows no thrust or a smaller negative contribution by inclining the lift vector backward (Rayner, 1988). The magnitude of the thrust is determined by the effective inclining angle and the lift magnitude (Muijres et al., 2012a,b; Tobalske, 2022). This can be approximately described by the following relation:
(3)

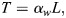
where *α*_*w*_ is the effective inclining angle of the lift vector.

In conclusion, weight support is achieved with the lift generated by the wings and thrust is achieved by inclining the lift forward due to flapping the wings. The magnitude of the lift is proportional to the area of the wings and the angle of attack, and it depends on the airfoil form (camber) and the airspeed. As described above, bats are able to control all of these factors by adjusting the shape and movement of the wings.

### Powered glide and glide with fixed wing posture

**Fig. 1.**
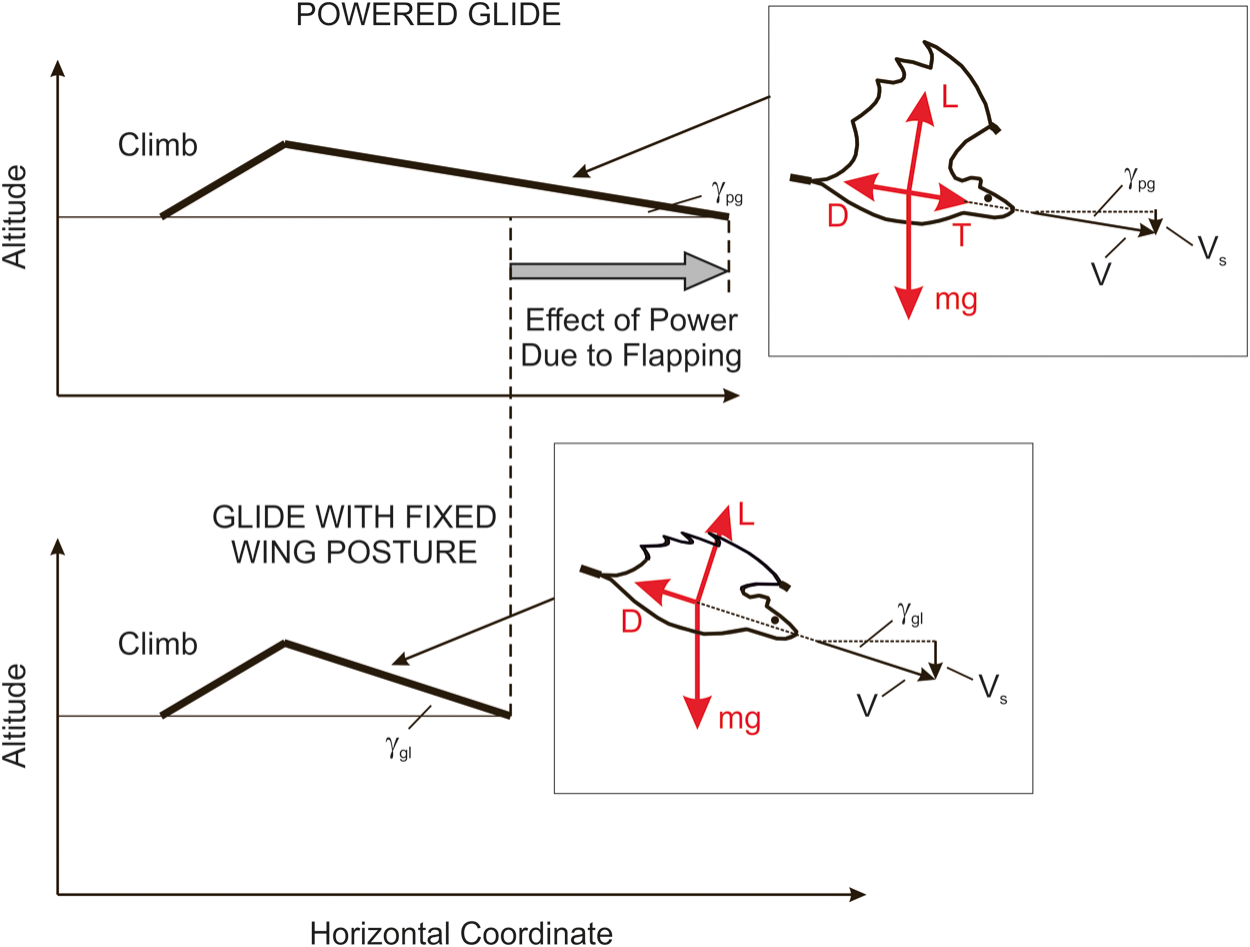
**Cycle of powered-gliding/climbing flight.** A cycle of powered-gliding/climbing flight is presented in the upper part, and a glide with a fixed wing posture in the lower part. The grey arrow shows how the distance covered in the powered glide is increased as compared to the glide with a fixed wing posture. Furthermore, the relationships concerning forces, speeds and descent angles are shown in boxes next to the respective trajectories. The forces are the drag *D*, lift *L* and thrust *T*. The speeds are the airspeed *V* and the descent rate *V_s_*. The descent angles are denoted by *γ*_*pg*_ and *γ*_*gl*_.

Powered-gliding/climbing flight is a flight mode that shows continual changes in altitude (Sachs, 2022). This flight mode is graphically addressed in Fig. 1 (upper part), which presents a cycle that consists of a climb and a powered glide. The powered glide is a descending flight technique with flapping of the wings. Accordingly, the powered glide is energetically supported by the mechanical power output of wing flapping.

A glide with a fixed wing posture is also presented in Fig. 1 (lower part). Comparing this with the powered glide, it becomes apparent how the power support due to flapping yields a decrease in the descent angle *γ*_*pg*_ of the powered glide against *γ*_*gl*_ of the glide with a fixed wing posture.

The flapping power in the powered glide is considered to be at an intermediate level. The term intermediate means that the generated power is smaller than the power required for flapping flight where the altitude is constant. The descent angle *γ*_*pg*_ can be controlled by adjusting the flapping power. The range of *γ*_*pg*_ extends from zero (flapping flight at constant altitude) to *γ*_*gl*_.

Concerning the wing shape change in flapping, gliding and powered-gliding flight, the unique wing characteristics and capabilities of bats enable an optimal adaptation of the wing shape for each of these flight modes, as described above. In flapping flight, the compliant bat wings with their highly elastic structure, enable a best possible morphological adjustment according to aerodynamic demands (Norberg, 1976; Hedenström and Johansson, 2015a,b). This holds for each down- and upstroke, which show different characteristics and demands. In gliding flight, the wings are kept outstretched in a fixed posture (Lindhe Norberg et al., 2000). Powered-gliding flight is similar to flapping flight but shows a change in wing kinematics and morphological adjustment, yielding a reduced power level (Sachs, 2022). The resultant force characteristics (shown in Fig. 1 for gliding and powered-gliding flight) can be described as follows:

- Flapping flight: lift provides weight support, and thrust equals drag.
- Gliding flight: lift provides weight support, and thrust is zero.
- Powered-gliding flight: lift provides weight support, and thrust is less than drag.

### Criterion for providing evidence of powered glide in bats

A procedure of finding out whether or not there is a powered glide is to compare the descent angle in an actual flight with the smallest descent angle that is aerodynamically possible with a fixed wing posture.

The descent angle in an actual flight, denoted by *γ*_*pg*_ and shown in Fig. 1, can be determined applying the following relation (Norberg, 1990):
(4)

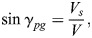
where *V*_*s*_ is the descent rate and *V* is the airspeed. The relationship between *V*_*s*_ and *V* is shown in Fig. 1 in the box next to powered glide.

There can be effects of moving air (wind, updraft, etc.) on *V_s_*. These are addressed in more detail below.

For determining the descent angle in a glide with a fixed wing posture, *γ*_*gl*_, reference is made to the equilibrium of the forces *D*, *L* and *mg* in gliding flight, as shown in Fig. 1 in the box next to the glide with a fixed wing posture. Examining this equilibrium, the following relations hold (Rosen and Hedenström, 2001):
(5)

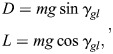
where *D* is the drag, *L* is the lift, *m* is the mass and *g* is the acceleration due to gravity. Eliminating the term *mg* in these relations, the following result can be obtained for the lift-to-drag ratio:
(6rma)

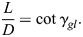
This result shows that *γ*_*gl*_ is the smaller the larger *L*/*D*. Thus, the smallest glide angle *γ*_*gl*,*min*_ can be achieved when gliding at the maximum lift-to-drag ratio (*L*/*D*)_*max*_, yielding:
(6rmb)

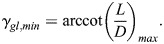
With reference to this result, the criterion for the existence of a powered glide can be formulated as:
(7)

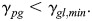


Five cases of bat flights will be examined with regard to this criterion below. Concerning the (*L*/*D*)_*max*_ values in the five cases, no data were available. Therefore, an alternative approach regarding that issue was developed.

For this purpose, available data on (*L*/*D*)_*max*_ of bats in respective papers were analysed. These data are presented in Table 1.

**Table 1.** Maximum lift-to-drag ratio (*L*/*D*)_*max*_ of bats used for reference purposes in analysing descent characteristics

Bat	Reference	Maximum lift-to-drag ratio (*L*/*D*)_*max*_
Palla's long-tongued bat (*Glossophaga soricina*)	Muijres et al. (2011a), Hedenström and Johansson (2015b)	7.54
Lesser long-nosed bat (*Leptonycteris yerbabuenae*)	Muijres et al. (2011a), Hedenström and Johansson (2015b)	6.93
Dog-faced bat (*Rousettus aegyptiacus*)	Pennycuick (1971)	6.8
Long-eared bat (*Plecotus auritus*)	Norberg (1976)	4

With reference to the range of the (*L*/*D*)_*max*_ data in Table 1, a value of (*L*/*D*)_*max*_=8 was chosen as adequate for the five cases of bat flights examined below. This is based on the assumption that the (*L*/*D*)_*max*_ values of the bats in the five cases are about (*L*/*D*)_*max*_=8 or smaller. For those cases where (*L*/*D*)_*max*_ is smaller than 8, the results using criterion Eqn 7 would be even more applicable.

The minimum glide angle associated with (*L*/*D*)_*max*_=8 is obtained from Eqn 6b to yield:

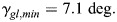


In this paper, five cases of bat flights are described that show continual altitude changes. These cases were chosen to cover a variety of flight types, extending from prey search to migratory flight. The goal is to provide evidence that there are powered glides in a wide range of different kinds of flight.

### Evidence of powered glide in bats

#### Case 1: aggregating for improving prey search

The first case concerns an aggregating of *Rhinopoma microphyllum* bats and how this can improve the search for prey or whether there are negative effects (Cvikel et al., 2015).

**Fig. 2.**
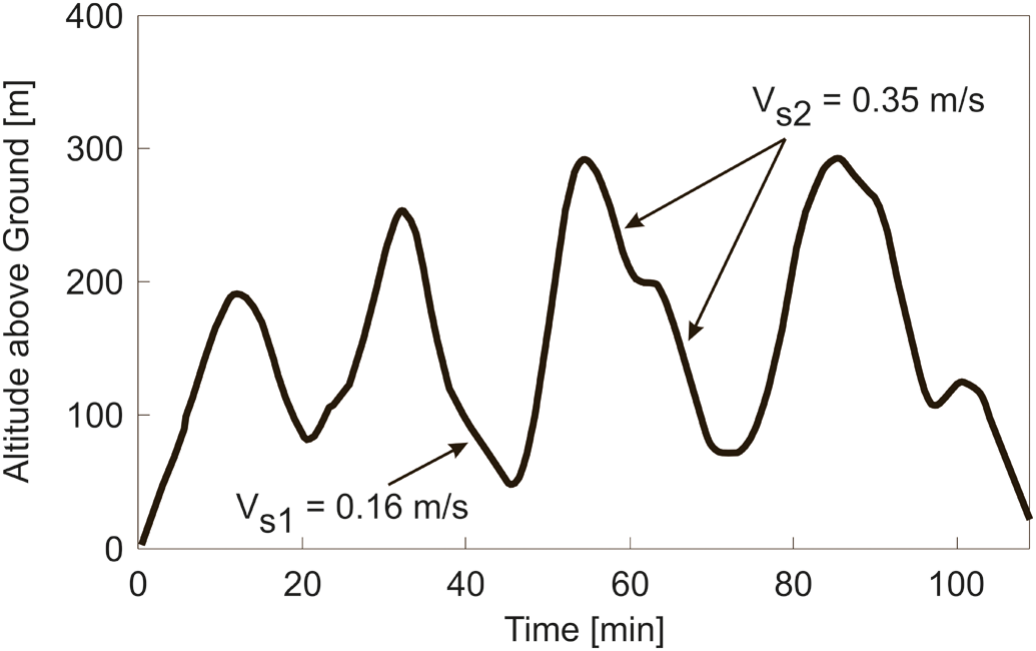
**Flight altitude data for a *R. microphyllum* bat.** The altitude (above ground) of a flight is presented along time for a *R. microphyllum* bat. Data adapted from Cvikel et al. (2015), where it was published under a CC-BY 4.0 license.

Flight altitude was determined using a global positioning system (GPS) data-logger. The GPS accuracy is ca. 8 m in the x-y plane and ca. 11 m in the z axis (altitude), according to (Cvikel et al., 2015, supplemental information). A flight trajectory is presented in Fig. 2 in order to show the descent characteristics and the altitude extensions of the cycles. Two descent rates are highlighted, yielding:

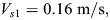
and:

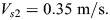
Furthermore, a value of *V*=5 m/s is given in (Cvikel et al., 2015).

Using Eqn 4 and the values of *V*_*s*1_, *V*_*s*2_ and *V*, the following results are obtained:

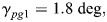
and

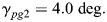
These results show that each of *γ*_*pg*1_ and *γ*_*pg*2_ is much smaller than *γ*_*gl*,*min*_=7.1 deg considered to hold for a descent with a fixed wing posture. This suggests that the descents are powered glides.

There are additional results in the paper referred to. It was found that all bats changed their altitude above ground (by at least 150 m). Furthermore, the need is expressed in Cvikel et al. (2015) that further study is required to understand this behaviour. This supports the above result that all bats performed powered glides.

#### Case 2: migratory flight

The second case of powered glides relates to migratory flights of bats. Reference is made to O'Mara et al. (2019b), which is concerned with three-dimensional tracks of common noctules (*Nyctalus noctula*).

Detailed three-dimensional representation of bat migration steps were derived using data from miniaturized barometric pressure radio transmitters and GPS positions of an airplane flying above each bat. Thus, the following results were obtained: *V*_*s*_=0.39±0.23 m/s and mean airspeeds *V*=7.2 –15.9 m/s (range: 0.45–35.6 m/s).

For determining *γ*_*pg*_, the following values were selected as representative:

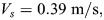
and

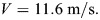
Using Eqn 4 and the values of *V*_*s*_ and *V*, the following result is obtained:

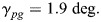


As *γ*_*pg*_=1.9 deg is much smaller than *γ*_*gl*,*min*_=7.1 deg, it can be concluded that the actual descents show a powered glide.

There is a statement in O'Mara et al. (2019b) that supports the powered glide result: “Most interesting were the unexpected altitudinal changes, which resembled those found in migrating thrushes (Bowlin et al., 2015)”.

It has been found that these unexpected altitudinal changes in migrating thrushes show powered glides (Sachs, 2022). This confirms the results of the above analysis on the existence of powered glides in common noctule bats.

**Fig. 3.**
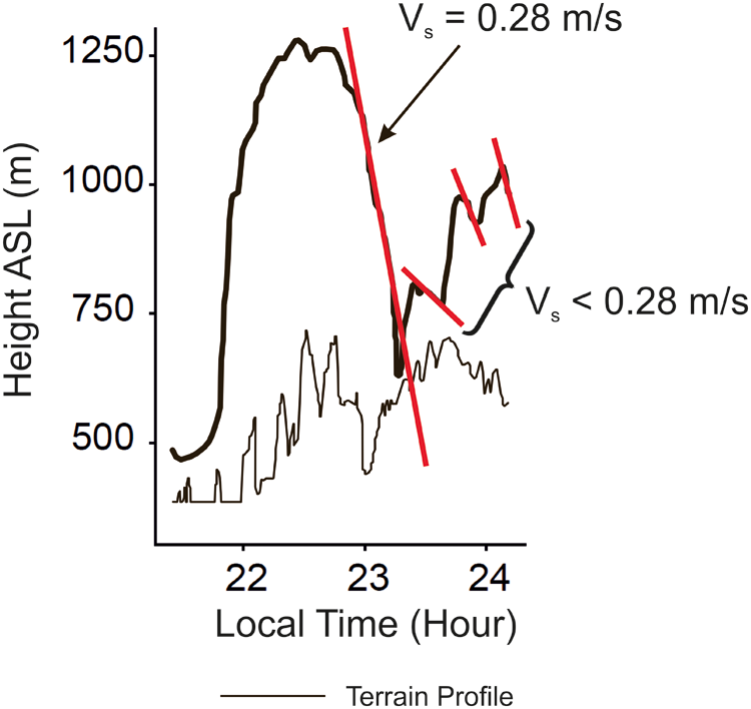
**Altitude profile of a migratory flight of a common noctule.** Altitude profile is plotted above the terrain profile of the ground level below. The terrain profile is indicated by the thin line. Data adapted from O'Mara et al. (2019b), where it was published under a CC-BY 4.0 license.

Additional to the above treatment, an excerpt of the results of O'Mara et al. (2019b) is plotted in Fig. 3 in order to show that portions of the flight altitude are much higher than the terrain profile of the ground level below. This suggests that orographic lift effects are minimal or not existent.

#### Case 3: low level flight

The third case concerns flights at low levels of the aerosphere (O'Mara et al., 2019a). Common noctules (*N. noctula*) were tracked in foraging flights and the use of vertical space was investigated.

Bats were tracked applying high-resolution atmospheric pressure radio transmitters that were used to determine the altitude. The transmitters had been calibrated for accuracy using the altimeter of a small aircraft (with *r*^2^=0.99). Thus, the following result was obtained: median descent rate *V*_*s*_=0.12 m/s (interquartile range: IQR=0.08–0.22). The airspeed showed values of *V*=6.0±2.1 m/s.

For determining *γ*_*pg*_, the following values were selected as representative:

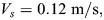
and

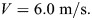


Using Eqn 4, the result concerning the descent angle is:

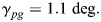
Comparing *γ*_*pg*_ with *γ*_*gl*,*min*_=7.1 deg suggests that the actual descent is a powered glide.

There is a further result in O'Mara et al. (2019a) according to which wingbeat frequency was independent from climbing or descending flight. This result is an indication of flapping in the descents. This confirms the above finding on the existence of powered glides.

#### Case 4: landscape-guided altitudinal flights

The fourth case concerns landscape-guided altitudinal flights (Roeleke et al., 2018). The flight behaviour of Theobald's tomb bats (*Taphozous theobaldi*) is investigated, and the role of ascents and descents is addressed.

Flights of bats were studied by equipping individuals with GPS tags. The GPS accuracy was ca. 8 m in the x-y plane and ca. 11 m in the z axis (Roeleke et al., 2016; Hurme et al., 2019). The following results were obtained: *V*_*s*_=0.46 m/s, with a range from *V*_*s*_=0.33 m/s to *V*_*s*_=0.69 m/s. Furthermore, it was found that the bats performed undulating ascending and descending flights for half of the overall flight time. This half was equally divided between ascents and descents.

It can be assumed that the upper value of the *V*_*s*_ range, *V*_*s*_=0.69 m/s, relates to a descent that shows a small power support due to flapping or a fixed wing posture. In this instance, it can be concluded that descents with smaller *V*_*s*_ values have a power support due to flapping. This suggests that these descents are powered glides.

**Fig. 4.**
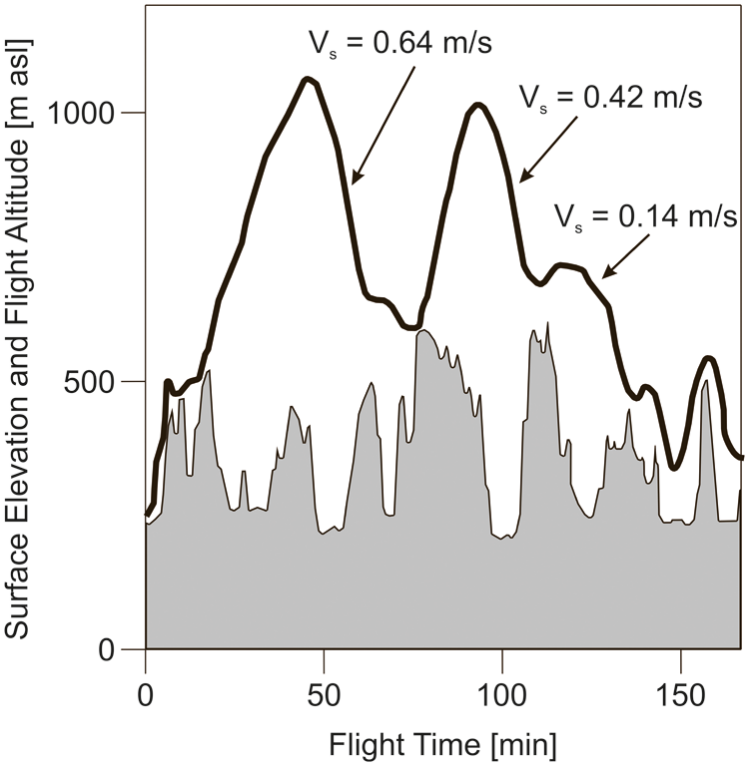
**Altitudinal flight of a *T. theobaldi*.** Flight altitude that reaches up to more than 1000 m is presented. The surface elevation below the flight altitude is also shown. Data adapted from Roeleke et al. (2018).

Exemplary for the results of Roeleke et al. (2018), a flight trajectory is presented in Fig. 4 to illustrate the descent characteristics and to show the altitude extensions of the cycles. Three descents are highlighted, showing the following values: *V*_*s*_=0.64 m/s, *V*_*s*_=0.42 m/s and *V*_*s*_=0.14 m/s.

In addition to the described results, there are two statements made in Roeleke et al. (2018) that are concerned with descent characteristics.

First statement: “The similarity of ascent and descent rates in *Taphozous theobaldi* might indicate that, during descent, bats engaged in powered flight and not gliding, since a descending glide might result in higher descent rates”. This confirms the above finding on the existence of powered glides.

Second statement: “In *T. theobaldi*, ascent and descent rates were not markedly different from each other, and they did not deviate largely from those observed in similar-sized birds (Bowlin et al., 2015)”. This also confirms that there are powered glides in *T. theobaldi*, since these birds show powered glides (Sachs, 2022).

#### Case 5: skyrocketing flight

The fifth case relates to skyrocketing flights of bats (Voigt et al., 2019). The flight behaviour of *T. theobaldi* was studied, and it showed that bats performed skyrocketing flights as a previously unrecognized behaviour.

The bats performed altitudinal flights with ascents, short horizontal flights and subsequent descents. The flight altitude was determined using a geolocator device. Ambient temperature and barometric pressure were converted into flight altitude using the hypsometric formula (Voigt et al., 2019; Liechti et al., 2018.). Accordingly, the accuracy concerning the altitude depends on actual atmospheric conditions in respect of that related to the hypsometric formula.

**Fig. 5.**
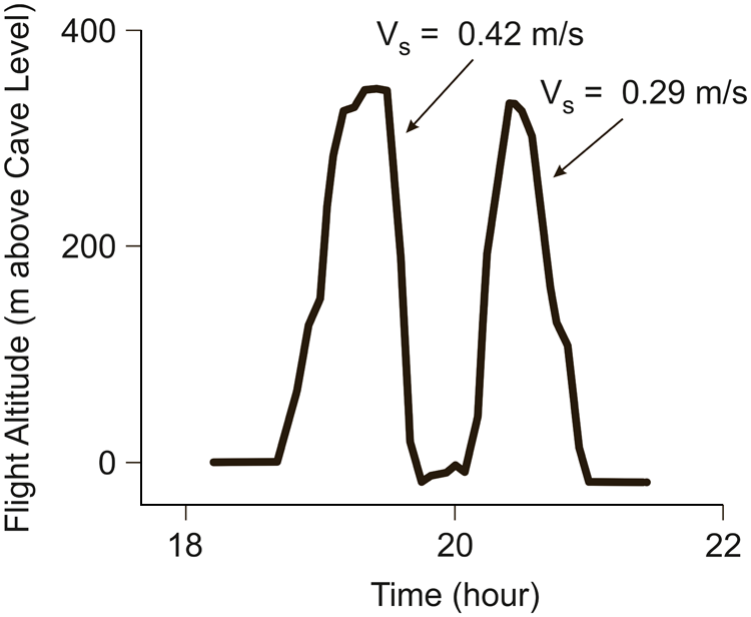
**Altitude profile of a flight of a *T. theobaldi*.** The flight altitude above cave level is presented. Data adapted from Voigt et al. (2019).

Results are presented in Fig. 5 to illustrate the descent characteristics and the altitude extensions. Two altitude cycles showing sink rates of *V*_*s*_=0.29 m/s and *V*_*s*_=0.42 m/s are presented. Assuming that the aerodynamics and mass properties of the bats are comparable to those of case 4 (Theobald's tomb bats in both cases), these sink rates indicate that the descents are powered glides.

#### Effects of moving air

The speeds *V*_*s*_ and *V* are used in Eqn 4 for determining *γ*_*pg*_. Both *V*_*s*_ and *V* basically relate to still air conditions.

The measured value of *V*_*s*_ can differ from the value in still air, depending on moving characteristics of the air. With regard to that issue, the following relationships are considered to hold:

(a) Wind (headwind, tailwind, crosswind): regarding the existence of wind scenarios, the measured value of *V*_*s*_ is equal to the value in still air, i.e. *V*_*s*_ is independent of whether or not there is a (horizontal) wind.
(b) Downdraft: the measured value of *V*_*s*_ in a downdraft is larger than the value in still air. This means that if the criterion relation *γ*_*pg*_<*γ*_*gl*,*min*_ holds for a measured *V*_*s*_ value in a downdraft, it would hold all the more for still air conditions.
(c) Thermal: in thermals, the measured value of *V*_*s*_ is smaller than the value in still air. However, thermals play a minor role for the nocturnal flights of bats. This is because the related energy sources may not be available at night due to the lower thermal potential in the nocturnal atmosphere (O'Mara et al., 2021).
(d) Orographic lift: this exitsts in terms of an updraft at the windward side of mountains or hills, implying that there is a downdraft at the lee side. Thus, a descent in the updraft would be associated with a climb in a downdraft for undulating flights with continual altitude changes. This is disadvantageous from a flight performance viewpoint so that it may be avoided by bats. Considering a descent in the downdraft at the lee side, the relation between the measured value of *V*_*s*_ and the value in still air described in b (above) holds. A further aspect regarding orographic lift is that this energy source may be not available for nocturnal flights of bats because of the difficulty of locating such uplift generating features at night (O'Mara et al., 2021).

The other quantity used in Eqn 4 is the airspeed *V*. This quantity, which describes the movement of a bat relative to the air, is independent from the wind and other moving air characteristics.

#### Conclusion

Five cases covering a variety of flight types were analysed with regard to descent characteristics. It was shown that there is power support due to flapping. This suggests that the descents are powered glides.

Furthermore, there is an aspect of general nature. This aspect is addressed by a statement in Voigt et al. (2019) that is concerned with the general existence of continual altitude changes: “Short altitudinal flights with fast ascents and descents … may present a so far unrecognized flight behaviour of bats that may be more widespread among other aerial insectivores”.

## DISCUSSION

### Flight mechanics of powered-gliding/climbing flight

The flight mechanics of powered-gliding/climbing flight are related to the energy characteristics of the motion. The energy characteristics regarding the climb (Fig. 1) concern the mechanical power output of wing flapping, the potential energy gain and the energy loss due to drag. The energy characteristics regarding the powered glide (Fig. 1) concern the use of the potential energy gain in the climb, the mechanical power output of wing flapping and the energy loss due to drag. It is assumed that the powered glide and the climb are motions of a long-term nature. Short-term, unsteady effects like the transition between climbing and gliding phases, are another type of motion and are considered of secondary importance.

As a starting point of the modelling approach, the power relation governing the climb (Fig. 1) was used. With reference to Hedenström and Alerstam (1992), this relation can be expressed as:
(8rma)

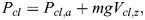
where *P*_*cl*_ is the mechanical power output due to flapping, *P*_*cl*,*a*_=*D*_*cl*_*V*_*cl*_ is the aerodynamic power with drag *D*_*cl*_ and airspeed *V*_*cl*_, and *V*_*cl*,*z*_ is the climb rate. Subscript *cl* is used to denote the climb.

The power relation governing the powered glide (Fig. 1) can be described in a manner analogue to Eqn 8a. This is because the powered glide can be regarded as a powered flight with a negative vertical speed (*V*_*pg*,*z*_<0). Thus:
(8rmb)

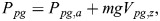
where *P*_*pg*_ is the mechanical power output due to flapping, *P*_*pg*,*a*_=*D*_*pg*_*V*_*pg*_ is the aerodynamic power with drag *D*_*pg*_ and airspeed *V*_*pg*_, and *V*_*pg*,*z*_ is the descent rate. Subscript *pg* is used to denote the powered glide.

There is a fuel consumption in both the climb and the powered glide because wing flapping requires a mechanical power output from the muscles. This can be modelled by introducing the relation for the rate of the fuel consumption:
(9)

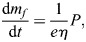
where *m*_*f*_ is the fuel consumption, *e* is the energy density of fuel and *η* is the conversion efficiency of chemical to muscular work (Alerstam and Hedenström, 1998).

The performance problem is in determining the fuel consumption per range. Due to the periodic character of powered-gliding/climbing flight, it is sufficient to deal with a single cycle for solving this problem.

Using Eqn 9, the fuel consumption of a cycle is obtained as:
(10)

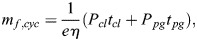
where *t*_*cl*_ is the time relating to the climb and *t*_*pg*_ is the time relating to the powered glide. With reference to Eqn 8a and 8b, this relation can be rewritten as:

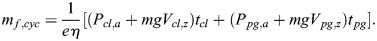
The terms *V*_*cl*,*z*_*t*_*cl*_ and *V*_*pg*,*z*_*t*_*pg*_ relate to the altitude extensions of the climb and the powered glide *h*_*cl*_ and *h*_*pg*_, yielding *h*_*cl*_=*V*_*cl*,*z*_*t*_*cl*_ and *h*_*pg*_=*V*_*pg*,*z*_*t*_*pg*_. Because of *h*_*pg*_=−*h*_*cl*_, the following relation holds: *V*_*cl*,*z*_*t*_*cl*_+*V*_*pg*,*z*_*t*_*pg*_=0. Taking this into account, the fuel consumption of a cycle can be expressed as:
(11)

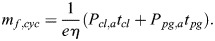
The aerodynamic power terms *P*_*cl*,*a*_ and *P*_*pg*,*a*_ and the underlying drag terms are treated as constant. This is in accordance with the long-term nature of the motion outlined above.

The distance covered during a cycle consists of the contributions of the climb and the powered glide. It is assumed that the flight path inclination is very small. Thus, the distance covered during a cycle can be described by:
(12)

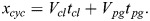


The performance criterion of the problem under consideration is the fuel consumption per range. This can be formulated as:
(13)

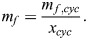
The goal of the flight optimization is to minimize the performance criterion.

Results of generally valid nature can be achieved by a nondimensionalization and normalization of Eqn 13 and the underlying mathematical relations. This includes the use of the power, speed and time terms 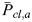, 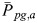, 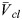, 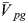, 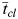 and 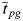. Details of the related procedure are described in the Materials and Methods.

According to this treatment, the following relation for the nondimensional and normalized form of the fuel consumption per range can be derived:
(14)

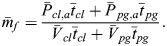


### Minimum fuel consumption and fuel saving enabled by powered-gliding/climbing flight

The fuel consumption per range as given by 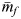 in Eqn 14 is the appropriate quantity for describing the fuel saving enabled by powered-gliding/climbing flight. The solution regarding the problem of fuel saving is to determine the minimum of 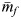. For this purpose, the mathematical relations underlying 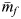 in Eqn 14 – including those described in the Materials and Methods – are used in an optimisation procedure. The goal is to determine the minimum fuel consumption per range.

**Fig. 6.**
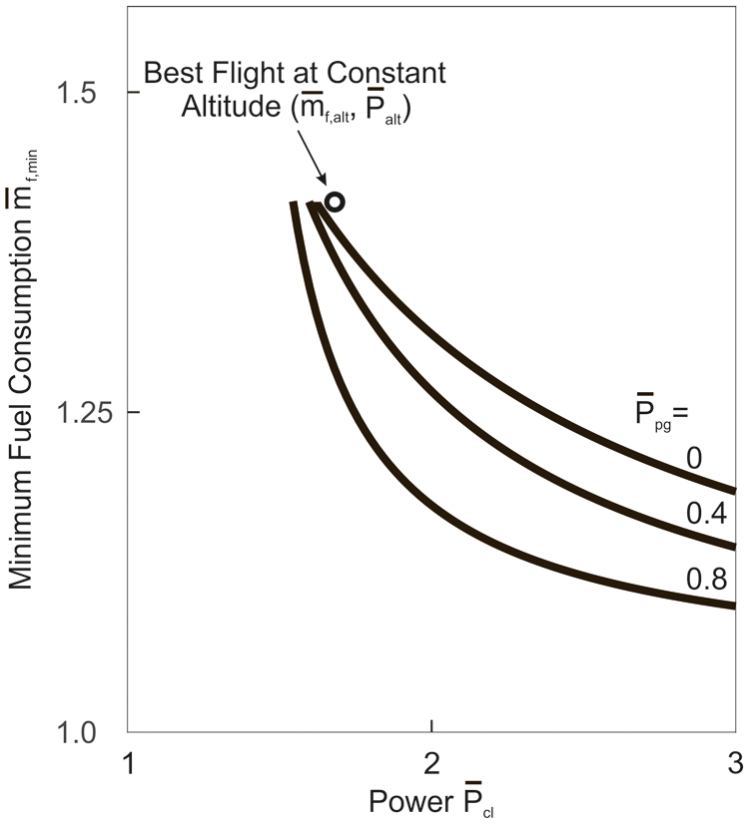
**Minimum fuel consumption per range achievable with powered-gliding/climbing flight for bats.** The minimum fuel consumption per range, 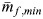, is presented dependent on the power in the climb phase, 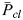. The effect of the powered glide is shown using several 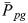 power values. Basically, the 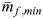 curve shows a continual decrease with 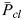 and 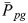. The decrease of 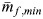 is strongest in the initial segment of the 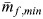 curve, with great effects due to each of 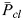 and 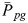. The minimum fuel consumption per range of the flight at constant altitude, 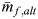, and the associated power, 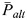, are also presented and referred to as best flight at constant altitude. A comparison shows that powered-gliding/climbing flight enables a fuel saving in the entire 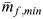-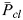 field.

Results obtained from this optimisation procedure are presented in Fig. 6 where the fuel consumption of powered-gliding/climbing flight for bats is shown. The minimum of the fuel consumption per range is denoted by 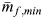. This is presented dependent on the power in the climb, 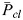. The effect of the power in the powered glide, 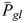, on 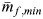 is also presented to show the related fuel saving potential. There are two basic results: 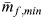 decreases with an increase of 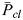 and with an increase of 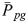. The effect of each of 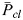 and 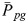 on 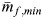 is strong, and the decrease of 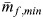 with both power quantities is continuous.

Due to the nondimensional and normalized form of 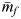, there is no explicit dependence of 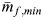 on bat properties concerning aerodynamics, size and mass. Accordingly, the 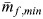 curve shown in Fig. 6 is independent of the properties of a specific bat. This means that the result presented in Fig. 6 is generally valid. For obtaining results holding for a specific bat, 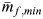 can be transformed back into the respective dimensional and not-normalized form using the corresponding relations in the Materials and Methods. For this procedure, the actual bat values concerning aerodynamics, size and mass have to be applied. Thus, the minimum fuel consumption per range can be determined for the respective bat.

To assess powered-gliding/climbing with regard to its fuel saving potential, a comparison is made with the flight at constant altitude that is classically considered as the best flight mode that requires the lowest fuel consumption (Pennycuick, 1975). Therefore, the flight at constant altitude is also addressed in Fig. 6. This is referred to as best flight at constant altitude, where 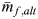 is the minimum fuel consumption per range of the flight at constant altitude and 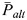 is the associated power (with 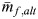 and 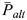 assigned to the respective ordinate and abscissa values). The optimization of the flight at constant altitude for determining 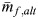 and 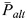 is described in the Materials and Methods.

The comparison presented in Fig. 6 shows that 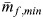 is smaller than 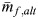. This is valid for all 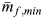 values. Thus, powered-gliding/climbing flight enables a fuel saving in the entire 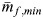-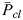 field. The fuel saving increases with the power in the climb, 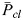. This holds true also for the effect of the power in the powered glide, 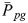.

An important finding regarding the fuel saving due to 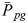 concerns the initial segment of the 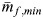 curve (at the left). As can be seen in Fig. 6, the gradient of the 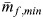 curve is strongest in this segment, to the effect that a rapid decrease of 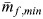 takes place. Thus, powered-gliding/climbing flight yields a substantial fuel saving in the initial segment of the 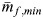 curve, where the required power effort concerning 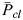 is lowest. A substantial contribution to this fuel saving effect is due to 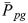, as evidenced by the overproportional decrease of 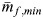 with 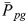.

The described fuel saving effect in the initial segment of the 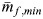 curve is an important advantage for bats. This means that a bat does not have to apply a higher flapping power in powered-gliding/climbing flight than in the flight at constant altitude in order to achieve a fuel saving. Rather, it is possible for a bat to achieve a fuel saving (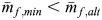) even with a smaller flapping power than in the flight at constant altitude (i.e. in the region where 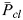 is smaller than 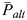).

The case 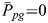 of powered-gliding/climbing flight shown in Fig. 6 is of special interest. This case is a glide with a fixed wing posture. There are two aspects addressed in the following.

The first aspect is relating to the flight performance in terms of the achievable fuel saving. The result presented for 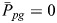 in Fig. 6 shows that a considerable fuel saving can be achieved even in this case, despite of the fact that no power support is provided in the glide.

The second aspect is that the case 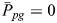 is equal to a flight mode that is known in the research on animal flight. This flight mode is termed flap-gliding flight (Tobalske and Dial, 1994; Rayner et al., 2001; Tobalske, 2010; Muijres et al., 2012a; Sachs, 2015b). It represents a prestage of powered-gliding/climbing flight. Flap-gliding flight shows the same flight characteristics as the case 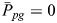 in terms of a climb with wing flapping and a glide with a fixed wing posture. Results presented in the literature show that flap-gliding flight yields fuel savings comparable to the case 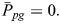 In the light of the equality of these two flight modes, powered-gliding/climbing flight can be considered as an extension of flap-gliding flight.

### Physical explanation for fuel saving by powered-gliding/climbing flight

The result presented in Fig. 6 shows that powered-gliding/climbing flight enables a substantial fuel saving. The powered glide has an essential effect on achieving the fuel saving, as evidenced by the influence of 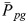 in that figure.

To understand the reason for the powered glide effect on fuel saving, the underlying aerodynamics will be discussed in the following. Thus, a physical explanation can be provided for the drag reduction by the powered glide as the basic reason for the fuel saving shown in Fig. 6. The aerodynamic treatment of this drag reduction is concerned with the vortex configuration existing in that flight mode.

**Fig. 7.**
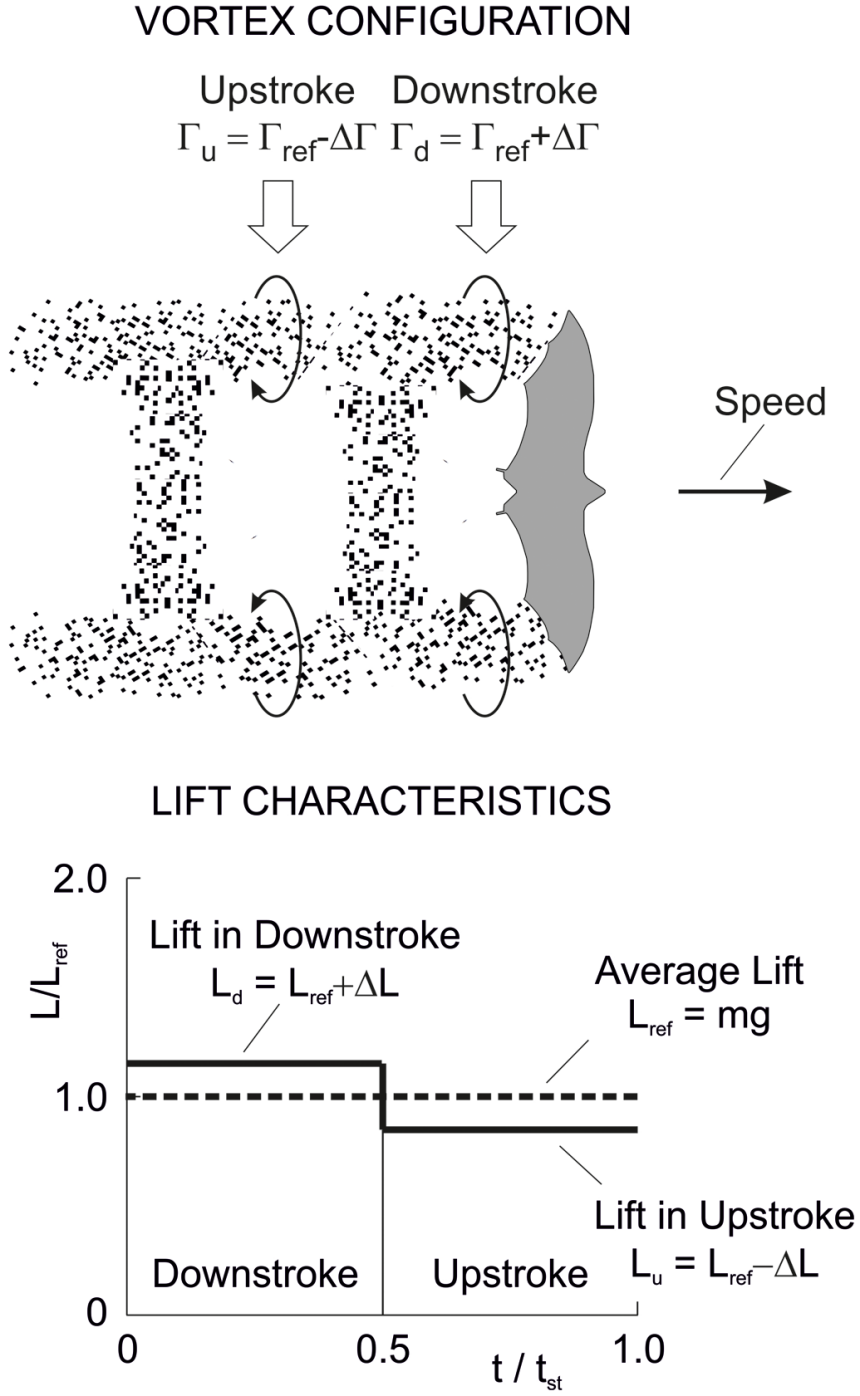
**Vortex configuration and lift characteristics of the powered glide.** It is assumed that the circulation is Γ_*ref*_+ΔΓ in the downstroke and Γ_*ref*_−ΔΓ in the upstroke. The lift is shown dependent on the time for the length of a wing stroke *t*_*st*_. According to the vortex configuration, the lift is *L*_*d*_=*L*_*ref*_+Δ*L* in the downstroke and *L*_*u*_=*L*_*ref*_−Δ*L* in the upstroke. The value of Δ*L* can be freely selected. It is assumed that the times of the down- and upstroke are equal.

The vortex configuration and the associated lift characteristics of the powered glide are presented in Fig. 7. The circulation is supposed to be Γ_*d*_=Γ_*ref*_+ΔΓ in the downstroke and Γ_*u*_=Γ_*ref*_−ΔΓ in the upstroke (subscript *ref* denotes an average, here and in the following). Correspondingly, the lift relations in the down- and upstroke are *L*_*d*_=*L*_*ref*_+Δ*L* and *L*_*u*_=*L*_*ref*_−Δ*L*. The value of the term ΔΓ and of the associated term Δ*L* can be freely selected. This holds for the range between the lower limit Δ*L*=0 and the upper limit Δ*L*=*L*_*ref*_.

Taking Δ*L* into account for the lift *L*_*pg*_ generated in the powered glide, the following relation holds in terms of the average lift of the entire wing stroke (providing weight support *L*_*pg*_=*mg*):
(15)

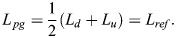


The increase of the lift by Δ*L* in the downstroke compared to the reference case *L*_*ref*_ suggests that there is an increase of the induced drag. Correspondingly, the decrease of the lift by –Δ*L* in the upstroke yields a decrease of the induced drag. Concerning these effects, it is assumed that there is a quadratic relation between the induced drag and the lift (according to corresponding drag-lift relations described in the Materials and Methods). This is used for deriving an estimate of the induced drag of the powered glide. Thus, the following result can be obtained for the induced drag in the downstroke:
(16rma)

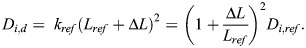
By analogy, the induced drag in the upstroke can be obtained as:
(16rmb)

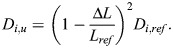
Using Eqn 16a and 16b, the induced drag of the powered glide can be determined to yield:
(17)

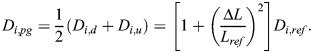
This relation is the induced drag underlying the result presented in Fig. 6.

Comparing *D*_*i*,*pg*_ with the induced drag *D*_*i*,*fl*_=2 *D*_*i*,*ref*_ in flapping flight (described in the Materials and Methods) and noting that Δ*L* is smaller than *L*_*ref*_ (i.e. Δ*L*/*L*_*ref*_ <1), the following result is obtained:
(18)

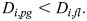
This means as a basic finding that the powered glide shows a smaller induced drag than flapping flight.

An important aspect is that Δ*L* can be freely selected (Fig. 7). Hence, *D*_*i*,*pg*_ of the powered glide can be influenced to reach a wanted value substantially smaller than *D*_*i*,*fl*_ of flapping flight. For example, a value of Δ*L*=0.3 *L*_*ref*_ yields:

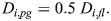
Thus, there is a substantial decrease of the drag in the powered glide compared to flapping flight.

The physical reason why the powered glide requires lower circulation than flapping flight during the downstroke phase is that the drag can be decreased by this means (as described by Eqns 17 and 18). The lowering of the circulation in the downstroke has to be compensated by generating a corresponding circulation in the upstroke in order to keep the total circulation related to the wing stroke constant. This is necessary for providing weight support.

To sum up, the powered glide shows a smaller induced drag than flapping flight. Thus, the powered glide yields a decrease of the drag work for the same distance covered. This is a generally valid result that is in correspondence with Fig. 6.

### Further aspects of powered-gliding/climbing

#### Independence of minimum fuel consumption 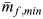 from altitude extension of cycle

The nondimensional and normalized form of 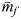, as described by Eqn 14, shows no dependency on the altitude extension of a cycle. The minimum fuel consumption per range, 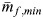, is obtained by minimizing 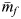. Thus, 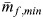 is also independent of the altitude extension of a cycle.

#### Minimum fuel consumption of consecutive cycles with different vertical extensions

It can be assumed that flights performed as powered-gliding/climbing flight show consecutive altitude cycles with different vertical extensions of the climb and the powered glide. Such flight scenarios involving a considerable variation in the vertical extensions exist in Figs 2 to 5 presented above.

**Fig. 8.**
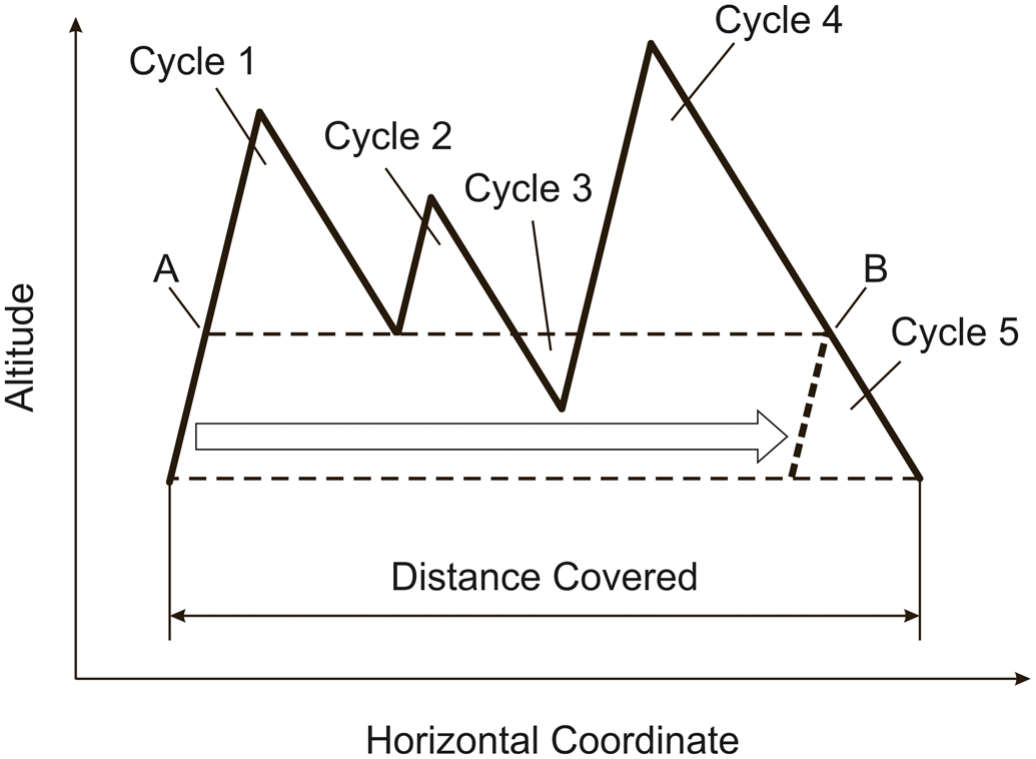
**Powered-gliding/climbing flight with consecutive cycles of different vertical extensions.** A series of consecutive altitude cycles with different vertical extensions of the climb and the powered glide is presented. It is presupposed that the power 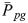 of the powered glides is the same in all cycles. Accordingly, it is presupposed that the power 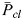 is also the same in all cycles. This is an optimality condition for achieving the minimum fuel consumption per range 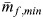 with regard to a series of consecutive cycles.

With regard to that behaviour, the problem arises how the minimum fuel consumption per range 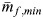 can be achieved in flights with varying altitude changes. This issue is graphically addressed in Fig. 8 where several consecutive cycles with different altitude extensions of the climb and the powered glide are presented, serving as an exemplary scenario. Two dashed lines are added, the lower one of which indicates the distance travelled of the whole cycle series. The upper dashed line is drawn through points A and B of the altitude curve. Thus, the altitude curve between these points can be subdivided into four individual cycles, marked by numbers 1 to 4. Each is a complete cycle in the sense that the altitude extensions of the climb phase and the powered glide phase are equal. Thus, each of the cycles 1 to 4 shows the minimum fuel consumption per range 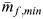. A fifth cycle is constructed by shifting the lower part of the leftmost climb (beneath point A) to the rightmost powered glide (at point B), as indicated by an arrow. Thus, a complete cycle termed number 5 is formed that is energetically equivalent to the others. As each of the cycles numbers 1 to 5 shows the minimum fuel consumption per range 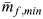, this holds true for the whole cycle series.

The result that 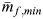 can be achieved for a series of differing consecutive cycles is an advantage for bats with regard to performing and controlling the flight. This is because they are not forced to repeat the same cycles. Instead, they are free in performing varying altitude cycles with different vertical extensions of the climb and the powered glide. This can be considered an advantage for the bats in achieving 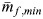 and an ease of control.

## MATERIALS AND METHODS

### Drag in non-flapping and flapping flight

The purpose of this section is to deal with the aerodynamic basis of non-flapping and flapping flight that are used for reference purposes regarding the powered glide. This is concerned with the vortex configurations existing in these flight modes. Based on the vortex configurations, the aerodynamic drag characteristics can be mathematically described.

#### Non-flapping flight

The generation of forces at the wing in the flight of bats can be described with the vortex theory of animal flight (Rayner, 1988; Norberg, 1990). This will be used in the following to show how the lift and the induced drag that are the determinative aerodynamic forces for the subject under consideration are generated.

**Fig. 9.**
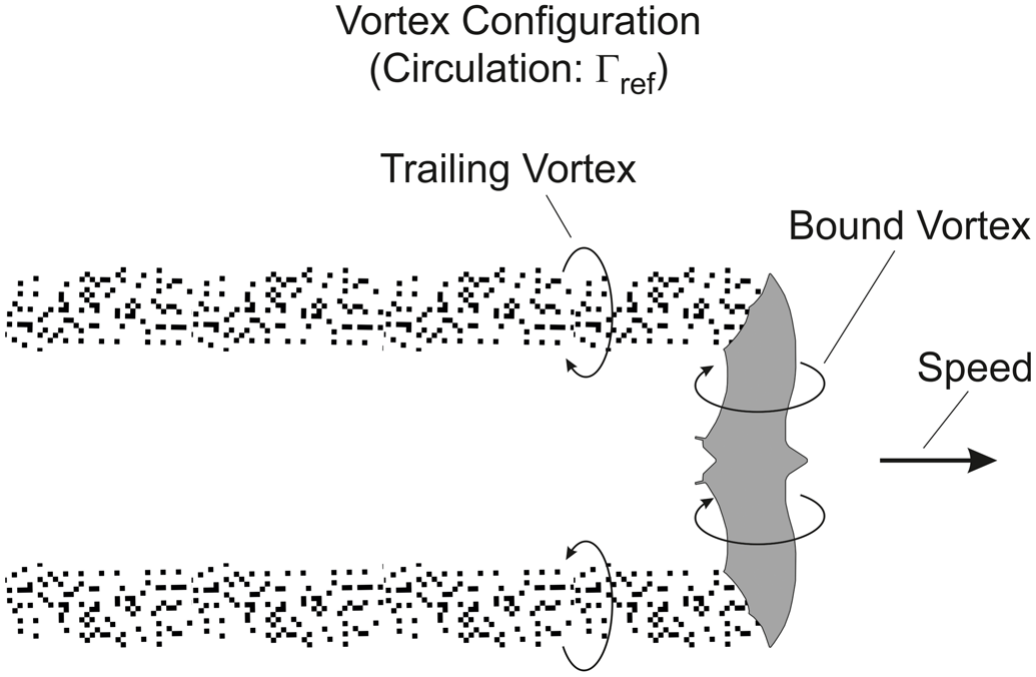
**Vortex configuration in non-flapping flight.** The vortex configuration considered to hold in non-flapping flight shows a bound vortex at the wing and two free wing-tip vortices behind the wing. The lift is generated by the bound vortex the circulation of which is supposed to be Γ_*ref*_. The two free vortices have the same circulation as the bound vortex.

At a wing moving through the air, rotational air flows are induced that take the form of vortices bound onto the wing and trailing from the wing tips (Rayner, 1988). This is graphically addressed in Fig. 9 for non-flapping flight. This vortex system causes a downward movement of the air behind the wing. As a reaction, there is a force generated at the wing in the opposite direction, yielding the aerodynamic lift (Rayner, 1988). The generation of the lift is associated with the bound vortex (Tobalske, 2022). The magnitude of the lift is proportional to the speed of the movement of the wing, *V*, and to the circulation of the bound vortex, Γ (Norberg, 1990). The direction of the lift is perpendicular to the local flow at the wing (Tobalske, 2022).

The relation between the lift and the circulation, *L*_*ref*_ and Γ_*ref*_, can be expressed for an elliptical lift distribution as (Norberg, 1990):
(19)

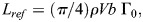
where *ρ* is the air density, *V* is the speed, *b* is the wing span, and Γ_0_ is the circulation at the middle of the wing span.

The lift provides weight support, yielding:
(20)

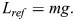


The lift acting at the wing is tilted backward due to the presence of downwash. The component of the lift acting parallel to the movement of the wing causes a retarding force termed induced drag. The induced drag associated with *L*_*ref*_ of Eqn 19 can be described by the following relation (Rayner, 2001; Norberg, 1990):
(21)

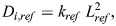
where

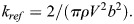


#### Flapping flight

**Fig. 10.**
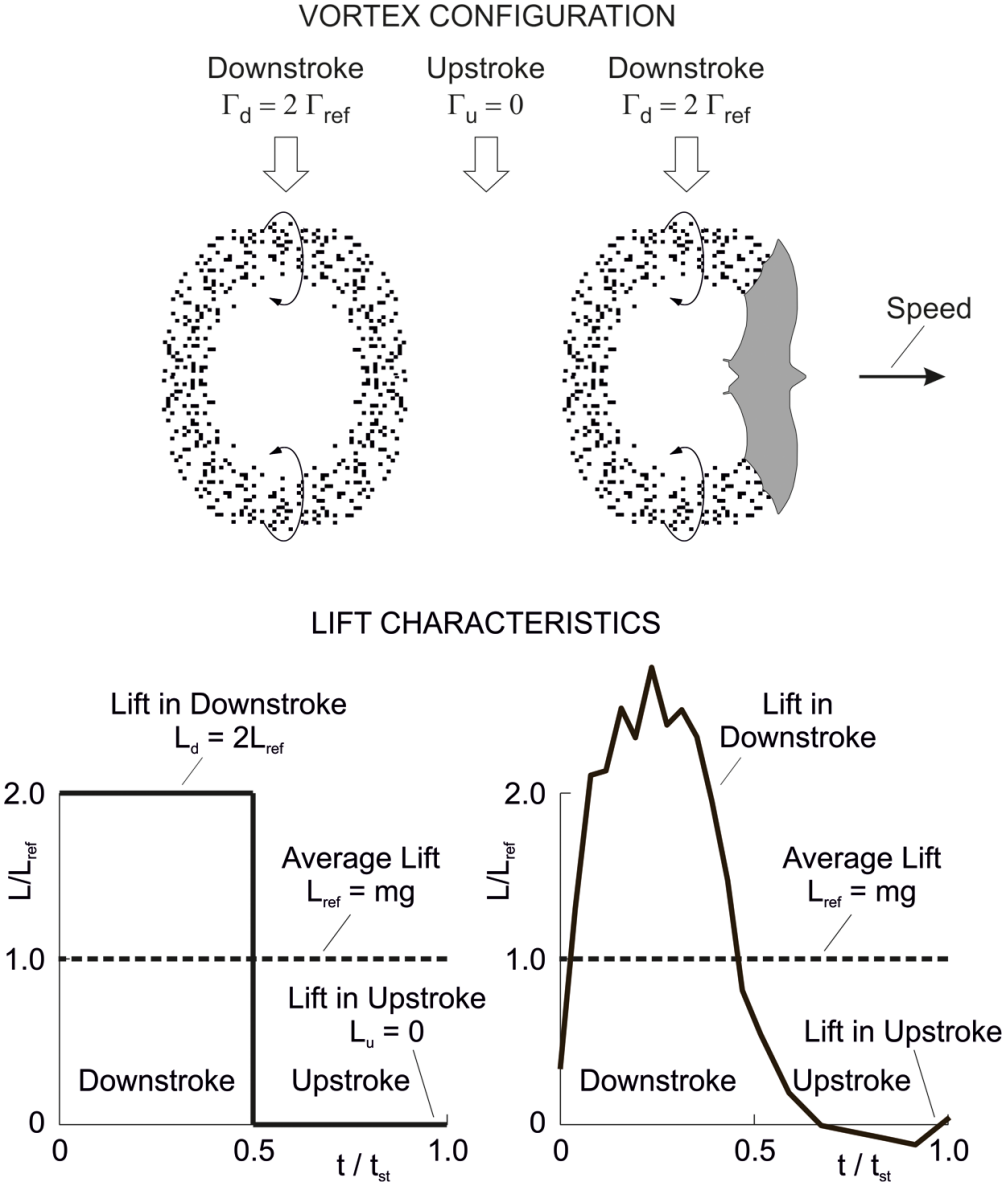
**Vortex configuration and lift characteristics in flapping flight.** The vortex configuration shows vortex ring elements that are associated with the downstroke. The gap between the vortex ring elements is related to the upstroke. At the left side of the bottom, the lift associated with the vortex configuration is shown dependent on the time for the length of a wing stroke *t*_*st*_. The lift is existent in the downstroke whereas the upstroke shows no lift. It is assumed that the times of the down- and upstroke are equal. At the right side of the bottom, the lift is presented using results from measurements. These results show that practically the entire lift is generated in the downstroke. The vortex configuration is a modified excerpt from Tobalske and Dial (1996), and the lift diagram at the bottom on the right is a modified excerpt from Muijres et al. (2011b), where it was published under a CC-BY 4.0 license.

Flapping flight which relates to the flight at constant altitude is graphically addressed in Fig. 10 that presents the vortex configuration and lift characteristics as shown and documented in related studies and papers. The vortex configuration shows vortex ring elements that are generated solely in the downstroke, and there is no vortex element related to the upstroke (Rayner et al., 1986; Rayner, 1988; Tobalske and Dial, 1996; Norberg, 1990; Tobalske, 2000; Hedenström, 2006). The vortex ring element of the downstroke has a circulation that is assumed to be Γ_*d*_=2 Γ_*ref*_. In the upstroke, the circulation is zero, Γ_*u*_=0. This means that the upstroke is aerodynamically inactive, showing no lift and no induced drag (only profile and parasite drag). The underlying biological mechanism is that the wingbeat kinematics show flexure and twisting of the wings during the upstroke and a reduction of the wing area (Norberg, 1976; Rayner et al., 1986).

The consequence of these vortex properties is that the downstroke must generate the whole lift for weight support, as shown in the left diagram at the bottom of Fig. 10. This is because the downstroke lift has to compensate for the zero-lift gap *L*_*u*_=0 of the upstroke. Thus for the lift in the downstroke:
(22rma)

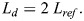
As a result, the following relation holds for the lift in flapping flight in terms of the average lift *L*_*fl*_ of the entire wing stroke (providing weight support *L*_*fl*_=*mg*):
(22rmb)

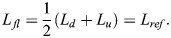


For dealing with the induced drag, reference is made to the generation of this kind of drag (Norberg, 1990). The presence of downwash and its effect on inclining the local flow in downward direction are of importance. Because of this, the lift vector is tilted backward. The tilting backward yields a lift component parallel to the speed so that a retarding force in terms of the induced drag is generated. The degree of tilting backward and the speed-parallel component can be regarded as proportional to the magnitude of the lift vector. This suggests that there is a quadratic relationship between the induced drag and the lift. It is assumed that this relationship holds for the flapping flight problem under consideration. Furthermore, it is assumed that the above factor *k*_*ref*_ can be used for an estimation of the quadratic relationship. Thus, for the induced drag of the downstroke:
(23rma)

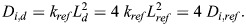
As a result, the following relation holds for the induced drag in flapping flight (average induced drag of the entire wing stroke with *D*_*i*,*u*_=0 in the upstroke):
(23rmb)

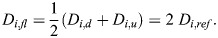
This means that there is an increase of the induced drag to twice the value of non-flapping flight as presented in Eqn 21.

Further to Fig. 10, the right diagram for the lift at the bottom depicts results from measurements of the flight of a lesser long-nosed bat *Leptonycteris yerbabuenae* (Muijres et al., 2011b). These results, which are presented for comparison purposes, show the temporal lift generation during the wing stroke where the lift *L* is referenced to the average *L*_*ref*_. A main aspect is that the lift is concentrated in the downstroke. This means that the lift is almost entirely generated in the downstroke, whereas the upstroke shows only an insignificant contribution. This corresponds with the lift characteristics presented in the left diagram.

Regarding the drag, a result of the lift concentration in the downstroke in the right diagram is that the induced drag is almost entirely generated in the downstroke. This corresponds with the induced drag holding for the case in the left diagram. This suggests that there is an accordingly large increase of the induced drag when compared with non-flapping flight.

### Nondimensionalization and normalization

The following quantities are used as references in the nondimensionalization and normalization procedure:
(24)

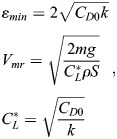
where 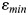 is the minimum drag-to-lift ratio (*C*_*D*_/*C*_*L*_)_*min*_ of the non-flapping reference case, 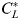 is the lift coefficient associated with 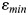, and *V*_*mr*_ is the maximum range speed (related to 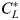).

With these references, nondimensional and normalized quantities (speed, drag, power, fuel consumption) are specified, as used for the results presented in the main text. The nondimensional and normalized quantities marked by an overline and associated base quantities are presented in the following, yielding speed of climb and powered glide:
(25rma)

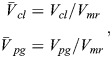


drag of climb and powered glide:
(25rmb)

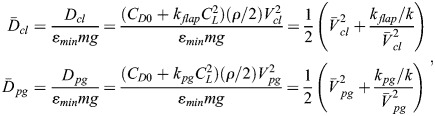


aerodynamic power of climb and powered glide:
(25rmc)

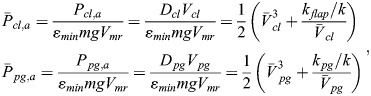
mechanical power output due to flapping of climb and powered glide:
(25rmd)

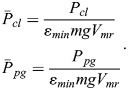
Using the aerodynamic power terms (Eqn 25c), the relation for the fuel consumption of a cycle (Eqn 11) can be expressed as:
(26rma)

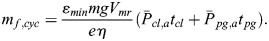
Using accordingly the speed terms (Eqn 25a), the relation for the distance covered during a cycle (Eqn 12) can be expressed as:
(26rmb)

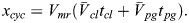
Applying Eqns 26a and 26b, the relation for the fuel consumption per range (Eqn 13) can be described by:
(26rmc)

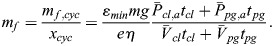


Furthermore, nondimensional times for the climb and the powered glide are introduced, yielding:
(26rmd)

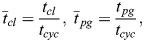
where *t*_*cyc*_ is the cycle time.

With the use of Eqns 26c and 26d, the nondimensional and normalized form of the fuel consumption per range (Eqn 14) can be obtained, yielding:
(26rme)

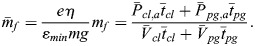


The lift dependent drag factor ratio is assumed to be *k*_*flap*_/*k*=2.

### Best flight at constant altitude

The force equilibrium in the flight at constant altitude can be described by:
(27)

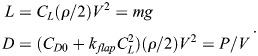


With reference made to the relation for the rate of fuel consumption, Eqn 9, and using the expressions *D*=(*D*/*L*)*mg*, the fuel consumption for the distance covered *x* is obtained as:
(28)

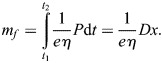


The minimum fuel consumption per range achievable with the flight at constant altitude, i.e. the minimum of *m*_*f*_/*x*, can be determined examining the relations described by Eqns 27 and 28. The following nondimensional and normalized relations for the minimum fuel consumption per range, denoted by 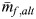, and the associated power, denoted by 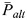, can be derived, yielding:
(29)

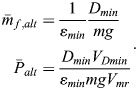
This is referred to as best flight at constant altitude.
